# Modeling of Memristive and Memcapacitive Behaviors in Metal-Oxide Junctions

**DOI:** 10.1155/2015/910126

**Published:** 2015-01-29

**Authors:** M. G. A. Mohamed, HyungWon Kim, Tae-Won Cho

**Affiliations:** ^1^Electronic Engineering Department, Chungbuk National University, Cheongju 361-763, Republic of Korea; ^2^Electrical Engineering Department, Faculty of Engineering, Minia University, Al-Minya 61111, Egypt

## Abstract

Memristive behavior has been clearly addressed through growth and shrinkage of thin filaments in metal-oxide junctions. Capacitance change has also been observed, raising the possibility of using them as memcapacitors. Therefore, this paper proves that metal-oxide junctions can behave as a memcapacitor element by analyzing its characteristics and modeling its memristive and memcapacitive behaviors. We develop two behavioral modeling techniques: charge-dependent memcapacitor model and voltage-dependent memcapacitor model. A new physical model for metal-oxide junctions is presented based on conducting filaments variations, and its effect on device capacitance and resistance. In this model, we apply the exponential nature of growth and shrinkage of thin filaments and use Simmons' tunneling equation to calculate the tunneling current. Simulation results show how the variations of practical device parameters can change the device behavior. They clarify the basic conditions for building a memcapacitor device with negligible change in resistance.

## 1. Introduction

Capacitor, resistor, and inductor are the three basic circuit elements which were invented by Ewald Georg von Kleist in 1745, Georg Simon Ohm in 1827, and Michael Faraday in 1831, respectively. These elements were constructed by experimental trials to observe the lumped behavior of relevant measurable electrical parameters across the devices [1]. Chua theoretically postulated the fourth passive element (memristor) in 1971 while trying to establish a missing constitutive relationship between the electrical charge and the magnetic flux [2]. Few years later, exactly in 1976, Chua and Kang extended the concept of the memristor (ideal memristor) to a broader class of memristive devices (generalized memristors) and systems proving that pinched hysteresis loop is their main feature [3]. Initial attempts had been done to build Chua's memristor with the help of active and passive elements [2, 3], while there was no physical realization for a passive physical device to show the memristive behavior because of the complexity involved in implementing memristors [4]. As a result memristors were almost forgotten, until Strukov et al., at Hewlett-Packard, accidently observed the memristive behavior in nanoscale cross-point resistive switches in their memory architecture [5, 6]. However resistive switches are not new [7]. They were first reported in 1960s with a simple metal-insulator-metal (MIM) structure [8–11] and developed in 1990s by including complex metal oxides [12, 13] and binary metal oxides [14, 15] as sandwiched materials. Recent reviews provide excellent broad overviews and a useful taxonomy of the proposed switching and conduction mechanisms of the various types of ReRAM [16–21]. Since then, memristors and memristive devices have been the focal point for researchers in material science, physics, and electronic engineering because of their passivity, nonvolatile properties, memory property, and nanodimensions.

Nanofeatures and ionic transport mechanism inherited in memristors introduce new challenges such as modeling, characterization, and system architectures. This interest is extended to other mem-elements [22] which include memcapacitors and meminductors to widen their application areas to memory devices and circuit design. These new devices can completely transform the current CMOS digital technology to a neuromorphic hybrid CMOS/mem-elements technology. They can open new areas for memory system design, multivalued logics, and artificial intelligence.

Memristive behavior was observed within the diffusion of metal cation and reduction/oxidation in metal-oxide junctions which have been addressed in the past two decades [17, 23–26]. Fabricated devices with different structure, sandwiched materials [27], and metal electrodes [28] have entailed either bipolar or unipolar switching behaviors. Therefore, extensive research has been done to better understand current memristor device characteristics during the past three years and hundreds of papers have been published in a short time. The underlying principle of the switching mechanism and previous published experimental work illuminate the possibility of existence of a memcapacitor device [24]. Memcapacitors and memristors are similar in their behavioral characteristics in that both depend on growth/shrinkage of filaments. In this paper, modeling techniques for the behavior of one of the most important mem-elements (memcapacitor) are proposed. Memcapacitors can be the basic element for future memory architectures.

This paper is organized as follows.  Section 2 gives brief explanation of mem-elements and compares their merits with basic passive elements.  Section 3 presents behavioral modeling techniques for the memcapacitor.  Section 4 discusses our memcapacitor physical model based on metal-oxide junction structure. Simulation results and discussions are given in Section 5, where the conditions for building a memcapacitor device with negligible memristive effects are highlighted. Finally, conclusions are presented in Section 6.

## 2. Mem-Elements

**Figure 1:**
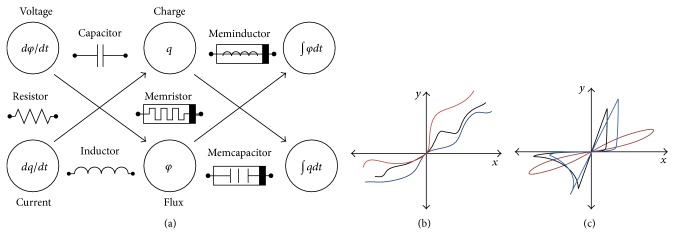
Classification of circuit elements (mem-elements and basic passive elements). (a) Illustration of circuit elements through a sequence of differentials of current and voltage. Mem-elements exhibit memory properties which convert nonlinear relation as in (b) to pinched hysteresis loop as in (c). (*x*, *y*) represents (*φ*, *q*), (*φ*, ∫*q* *dt*), and (*q*, ∫*φ* *dt*) for memristor, memcapacitor, and meminductor, respectively, in case of nonlinear relations in (b), while (*x*, *y*) represents (*v*, *i*) for memristor, (*q*, *v*) for memcapacitor, and (*φ*, *i*) for meminductor in case of pinched hysteresis loops in (c).

Memristive behavior has been observed through the investigation of nanostructures [6]. As highlighted by Chua and Kang in their classic paper, a memristor device has a relation between the first derivatives of current and voltage which changes the linear relation of a normal resistor to pinched hysteresis loop [3] as shown in Figure 1. Di Ventra et al. [22] extended the notion of memristive systems to capacitive and inductive elements setting up another group of elements “mem-elements”—memristor, memcapacitor, and meminductor. Mem-elements differ from basic passive elements in exhibiting memory property which converts the linear relation to a pinched hysteresis loop.

For better understanding, there is a generalized model which represents the behavior of mem-devices [22]. It is supposed that *x* denotes a variable describing the internal state of the system. *u*(*t*) and *y*(*t*) are any two complementary constitutive variables (i.e., current *i*, charge *q*, voltage *v*, or flux *φ*) denoting input and output of the system, and *g* is a generalized response. Then a general class of *n*th-order *u*-controlled memory devices is defined as

(1) $$\begin{array}{c} y\left(t\right)=g\left(x,u,t\right)u\left(t\right),\\ \dot{x}=f\left(x,u,t\right), \end{array}$$

where *f* is a continuous *n*-dimensional vector function. Figure 1 simplifies the relation of the three mem-elements to illustrate the definition of each one [29]. For example, the memcapacitor is a capacitor whose capacitance is defined as a state function of voltage or charge changing with time. In the same fashion, the memristor can be defined as a resistor whose resistance is defined as a state function of current or voltage changing with time, and the meminductor is an inductor whose inductance is defined as a state function of current or flux changing with time.

**Figure 2:**
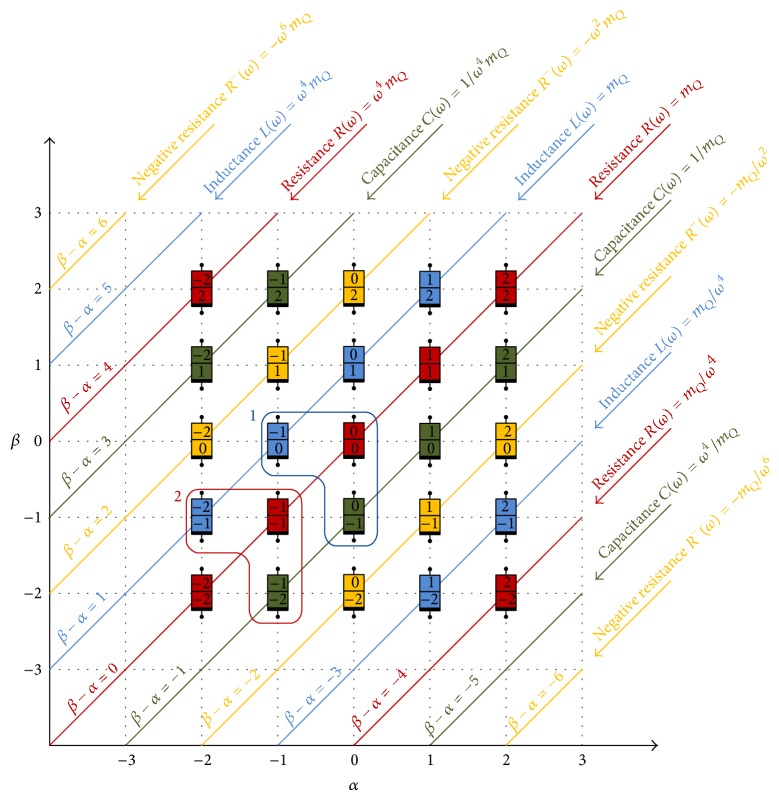
Periodic table of circuit elements [22]. Group 1 contains the three basic passive well known elements, while group 2 contains the basic mem-elements.

Figure 2 presents the periodic table of circuit elements to investigate all possible relations through a sequence of current derivatives and voltage derivatives. This periodic table was first introduced by [30]. Each device is named by (*α*, *β*) in which (*α*, *β*)-element represents an element with a relation between *α*-derivative of voltage *v* ^(*α*)^(*t*) and *β*-derivative of current *i* ^(*β*)^(*t*). This table shows an interesting property that all elements in the table show one of the following basic behaviors in certain fashion: resistive (positive or negative), capacitive, or inductive.

For instance, ideal resistor has a relation between voltage *v*(*t*) and current *i*(*t*), whereas ideal capacitor has a relation between first derivative of voltage *v* ^(1)^(*t*) and current *i*(*t*). In a similar fashion, ideal inductor has a relation between voltage *v*(*t*) and first derivative of current *i* ^(1)^(*t*); see group 1 of Figure 2. However mem-elements in group 2 show the following relations: memristor (−1, −1) shows a relation between first integration for current and first integration of voltage, memcapacitor (0, −1) shows a relation between first integration for current and voltage, and meminductor (−1,0) shows a relation between current and first integration of voltage.

## 3. Behavior Modeling of Memcapacitor

Capacitor is one of the basic elements, which was invented in 1745 by Ewald Georg after discovering that the charge can be stored by connecting high voltage generator to a volume of water. Nowadays the capacitor is the most important passive element in VLSI circuits especially because of parasitic capacitances and their huge effect on circuit behavior. Therefore it is often necessary to model variable parasitic capacitances in VLSI circuits.

### 3.1. Variable Capacitor Modeling

A capacitor with variable capacitance *C* which is represented by a function *f*_ctrl_ can be modeled as follows:

(2) $$\begin{array}{c} q\left(t\right)=Cv\left(t\right), \end{array}$$

(3) $$\begin{array}{c} i\left(t\right)=C\frac{dv\left(t\right)}{dt}, \end{array}$$

where *v*(*t*) is the applied voltage across the capacitor and *q*(*t*) is the charge accumulated over its terminals.

**Figure 3:**
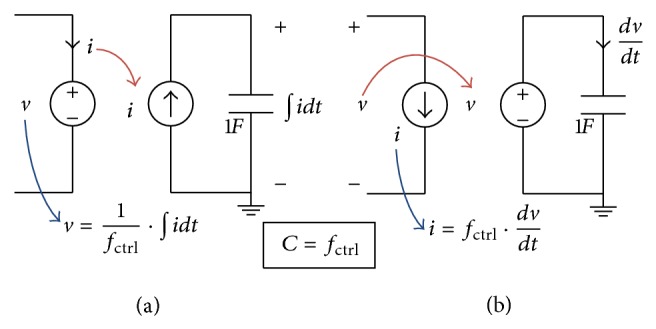
Schematic diagram of the variable capacitor model using a controlled voltage source and a separate integrator circuit to calculate charge by integration of current as in (a) or a controlled current source and a separate differentiator circuit to calculate the differentiation of voltage as in (b). The capacitance is considered to be controlled by a function *f*_ctrl_ which can be chosen to be dependent on the applied voltage, the passing charge, or any other variable.

Equations (2) and (3) illustrate the principles for building a variable capacitor model. The variable capacitor can be modeled in two ways: a voltage source which is controlled by integration of current as shown in Figure 3(a) or a current source which is controlled by differentiation of voltage as shown in Figure 3(b). In these modeling techniques of variable capacitor, separate capacitor circuits have to be included in order to determine the integration of current or differentiation of voltage.

### 3.2. Memcapacitor Modeling

Equations (1) give a general behavior for mem-elements. They can be rewritten to model memcapacitor in two ways as follows.

(1) Voltage-dependent memcapacitor:
  (4) $$\begin{array}{c} q\left(t\right)=C\left(x,v,t\right)v\left(t\right),\\ \dot{x}=f\left(x,v,t\right). \end{array}$$
(2) Charge-dependent memcapacitor:
  (5) $$\begin{array}{c} v\left(t\right)=C^{-1}\left(x,q,t\right)q\left(t\right),\\ \dot{x}=f\left(x,q,t\right). \end{array}$$

**Figure 4:**
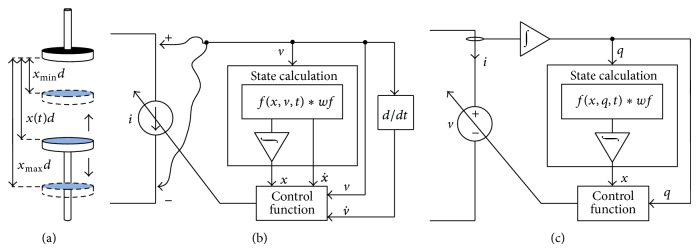
General behavioral model for a memcapacitor. (a) Device structure as a basic variable capacitor as published in [31, 32]. (b) A flow diagram of voltage-dependent memcapacitor model. (c) A flow diagram of charge-dependent memcapacitor model using a controlled voltage source. Other block diagrams are included to calculate the state and control functions in (b) and (c). The state calculation block diagrams use a function *f*(*x*, *v*, *t*) as in (b) or *f*(*x*, *q*, *t*) as in (c) which are multiplied to the window function *wf* and then fed to an integrator.

From (4)-(5), it is clear that memcapacitor is a voltage or charge controlled variable capacitor. A pinched hysteresis loop behavior between voltage and charge is also the main property of memcapacitor. Therefore memcapacitance can be easily represented by using the basic formula for a capacitor as by (2) and modeling it using a controlled voltage or current source. Their values are determined by an equation depending on charge or voltage as shown in Figure 4, and therefore they show the pinched hysteresis loop behavior.

Let us consider a simple structure for the memcapacitor device that consists of a dielectric material sandwiched between two metal plates: fixed and moving ones as shown in Figure 4(a) which was previously published by Biolek et al. [31, 32]. The device capacitance is given by

(6) $$\begin{array}{c} C=\frac{\varepsilon A}{x\left(t\right)d}, \end{array}$$

where *ε* is the permittivity of the gap material, *A* is the plate cross section area, *d* is the maximum separation between the two plates, *x*(*t*) is a positive state variable whose value is less than 1, and *x*(*t*)*d* is the dielectric thickness in a certain time *t*. The state variable *x*(*t*) defines the boundaries between 0 and 1 (or 0 < *x*_min⁡_ < *x*(*t*) < *x*_max⁡_ < 1).

#### 3.2.1. Voltage-Dependent Memcapacitor Model

For voltage-dependent memcapacitor, the change in the position of the moving plate is linearly proportional to the applied voltage over the memcapacitor device. The following equation is proposed to calculate the change in the separation of the two plates:

(7) $$\begin{array}{c} \dot{x}=-kv\left(t\right)f\left(x,v,t\right). \end{array}$$

To confine boundary conditions, the window function *f*(*x*, *v*, *t*) is included as done in the published work [6, 33]. The following window function insures the boundary conditions (0 < *x*(*t*) < 1) and depends on voltage:

(8) $$\begin{array}{c} f\left(x,v,t\right)=1-{\left(x\left(t\right)-\text{stp}\left(v\right)\right)}^{2P}, \end{array}$$

where *P* is a parameter that can control the nonlinearity of the window function and stp(*v*) is a step function which equals 1 for *v* > 0 and 0 for *v* ≤ 0. This window function limits the value of *x* between two values *x*_min⁡_ = 0 and *x*_max⁡_ = 1. However it can be modified to include variable boundary limits as follows:

(9) $$\begin{array}{c} f\left(x,v,t\right)=\delta -\vartheta {\left(x\left(t\right)-\text{stp}\left(v\right)\right)}^{2P},\\ \delta =x_{\max},\\ \vartheta =x_{\max}-x_{\min}. \end{array}$$

In the case of the voltage-dependent memcapacitor, we can use (2) and (6) to derive the model by using integration as follows:

(10) $$\begin{array}{c} \frac{d}{dt}\left(q\left(t\right)\right)=\frac{d}{dt}\left(\frac{\varepsilon A}{x\left(t\right)}v\left(t\right)\right), \end{array}$$

(11) $$\begin{array}{c} i\left(t\right)=\varepsilon A\left(\frac{\dot{v}\left(t\right)x\left(t\right)-v\left(t\right)\dot{x}\left(t\right)}{x^{2}\left(t\right)}\right). \end{array}$$

This way, we propose to model the memcapacitor device with a controlled current source to calculate (11) with introduction of other circuit components to get the state and voltage differentiation as shown in Figure 4(b).

#### 3.2.2. Charge-Dependent Memcapacitor Model

For a charge-dependent memcapacitor device, the moving plate is assumed to be linearly dependent on the amount of charge passing through the device

(12) $$\begin{array}{c} \dot{x}=-kq\left(t\right)f\left(x,q,t\right), \end{array}$$

where *f*(*x*, *q*, *t*) represents the window function that depends on the charge as follows:

(13) $$\begin{array}{c} f\left(x,q,t\right)=\delta -\vartheta {\left(x\left(t\right)-\text{stp}\left(q\right)\right)}^{2P}. \end{array}$$

By substituting (6) into (2), the charge-dependent memcapacitor can be described as follows:

(14) $$\begin{array}{c} v\left(t\right)=\frac{x\left(t\right)d}{\varepsilon A}\left(\int i\left(t\right)dt+q\left(0\right)\right). \end{array}$$

Therefore the charge-dependent memcapacitor device is modeled with a controlled voltage source with introduction of other circuit components to calculate its state and charge with current integration as shown in Figure 4(c).

**Figure 5:**
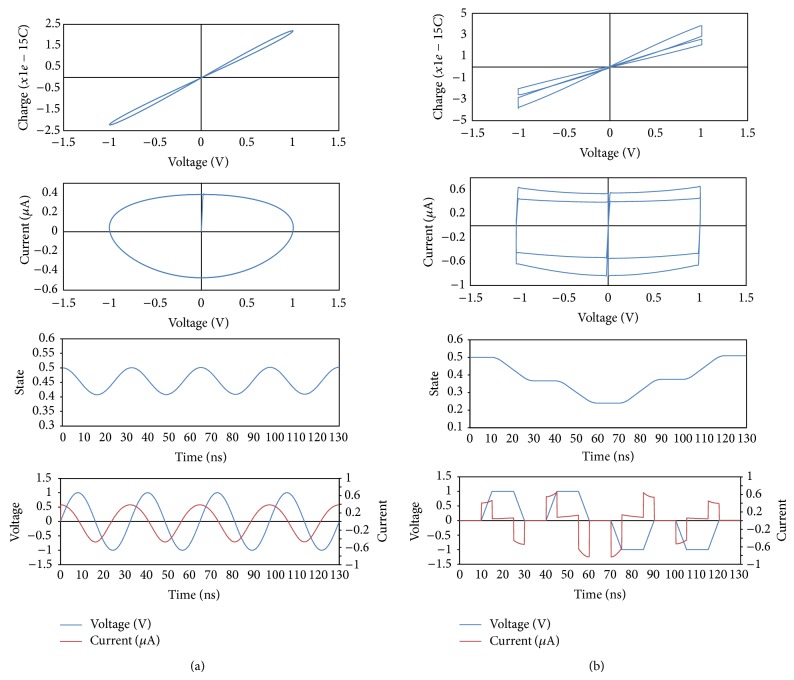
SPICE simulation results for a memcapacitor device using the proposed behavioral model with applying a sinusoidal voltage with 1 V amplitude as in (a) and a sequence of pulses with positive and negative polarities as in (b). In case of sinusoidal voltage in (a), the current is shifted by 90° and the device state changes smoothly in phase with applied voltage. In case of (b), the current almost equals zero when the voltage keeps constant, while it changes when there is a low-to-high transition or high-to-low transition. Device state also changes when the voltage is not zero. Current-voltage plot shows elliptic curve highlighting the capacitive behavior in (a) while it shows a pinched loop in (b) because the current and voltage drop to zero in the same time. The pinched hysteresis loops in current-voltage plots highlight device memory property in both cases. Simulation parameters: *x*_min⁡⁡_ = 0, *x*_max⁡⁡_ = 1, *A* = 20*E* − 20. *A* = 20*E* − 20, *d* = 20*E* − 10, *P* = 4, and *ε* = 10.

Figures 4(b) and 4(c) show the flow diagram for the voltage and charge-dependent memcapacitor models. They illustrate how (11) and (14) are used to build a memcapacitance model in a simple way. By simulating this model in SPICE with various voltage waveforms as shown in Figure 5, we indeed obtain a pinched hysteresis loop between voltage and charge ensuring that our proposed techniques accurately model the memcapacitive behavior. Whenever a sinusoidal voltage is applied as shown in Figure 5(a), the model generates a sinusoidal current with 90° phase shift and variable current amplitude ensuring that this device is a capacitor with a variable capacitance over time. The state plot also shows that the device state changes with time illustrating that the capacitance is not constant. However the current-voltage plots form Lissajous figures showing that it is a capacitor with different values of capacitances. Whenever a pulse-shaped voltage is applied as shown in Figure 5(b), the current increases in case of changing voltage from a state to the other and tends to zero in case of no change in voltage. The state is also changing with each pulse decreasing with positive voltage and increasing with negative voltage producing opposite changes in capacitance.

## 4. Physical Modeling of Metal-Oxide Junctions

This section discusses modeling of the metal-oxide junction taking into consideration the parasitic capacitance values between the two metal electrodes and thin films constructed within the insulator layer. In the past, metal-oxide junction has been considered as a common structure that shows memristive behavior [34]. Recently various materials have also been used to fabricate this device [23, 35]. Using the structure, some changes in dimensions and number of insulator layers sandwiched between metal electrodes have been examined [16]. It has been shown that these changes have large impact on the device behavior and so lead to capacitance changes as well as resistance changes [26]. As a result, the metal-oxide structures that show memristive behavior can also be used as memcapacitive devices.

**Figure 6:**
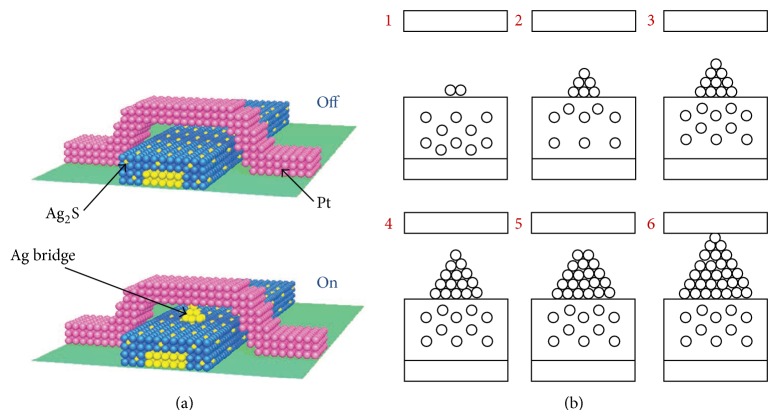
Gap-type ionic switch structure. (a) Device schematic formed in crossbar structure published in [23]. (b) Steps of filament growth through the junction (steps 1–4 show changes in filament length and cross section area, while steps 4-5 show changes in filament cross section area).

In this case, we suggest that the device has to be modified to keep high on/off resistances to eliminate memristive behavior. Filament growth/shrinkage through the device also has to be voltage or charge dependent as presented in (4)-(5) to keep the pinched hysteresis loop behavior. However, fabrication of a metal-oxide junction with an addition of multilayer sandwiched materials of high permittivity tends to have more prominent memcapacitive behavior. Therefore it can keep the resistance high, even with full filament growth.  Figure 6 shows the basic structure of this device and filament growth.

The gap-type ionic switch [23, 35] shows a good memristive structure with using Ag_2_S as a sandwiched material between platinum electrodes (Figure 6(a)). Modifying this device by changing the material or the gap size will greatly affect the device behavior [7]. Using a high-K dielectric may also help eliminate memristive behavior and clearly show the memcapacitive behavior.

Metal-oxide junction, shown in Figure 6, experiences two current components: tunneling current *i*_*t*_(*t*) and the capacitive current *i*_*c*_(*t*) [36]. It can be modeled by a controlled current source whose value equals a summation of both current components. Therefore modeling of such junctions goes through three steps of calculations: device state, capacitive current, and tunneling current.

### 4.1. Device State

Figure 6(b) shows steps of filament growth through the junction by ions migration causing filament growth/shrinkage that considerably affects device resistance and capacitance. The device passes through two steps of filament growth:

1. change in filament length and cross section area simultaneously (steps 1–4),
2. change in filament cross section area with constant length (steps 4–6).

Therefore it is essential to determine both filament length and cross section area. Assume that the gap separating top metal and filament has a length *x*(*t*)*d* and the cross section area of the filament is *m*(*t*)*A*. Here *d* is the thickness of the sandwiched layer that experiences filament growth and *A* is the cross section area of the device. It is known by [23] that the filament position is exponentially affected by tunneling current *i*_*t*_(*t*) as follows:

(15) $$\begin{array}{c} \frac{dx\left(t\right)}{dt}=Ke^{\left(\left(-E+Di_{t}\left(t\right)\right)/k_{B}T\right)}. \end{array}$$

Here *E* is the activation energy, *k*_*B*_ is Boltzmann constant, *T* is temperature, and *K* and *D* are the constants to scale the nonlinearity. The differences of the derivative of filament growth and shrinkage rates yield different coefficients, that is, *K*_*s*_ and *D*_*s*_ for shrinkage and *K*_*g*_ and *D*_*g*_ for growth:

(16) dxtdt=−Ksstpittexp⁡⁡−E+DsittkBT+Kgstp−ittexp⁡⁡−E−DgittkBT.

To impose boundary conditions on (15) and (16), a window function *f*(*x*, *i*_*t*_, *t*) is introduced to the right hand side of these equations. An exponential function is proposed for the boundary conditions to reflect the exponential nature of the filament growth as follows:

(17) fx,it,t=δx−ϑxstp−itt−xt2∗exp⁡⁡−Pstpitt−xt2,

(18) $$\begin{array}{c} \delta_{x}=x_{\max}, \end{array}$$

(19) $$\begin{array}{c} \vartheta_{x}=x_{\max}-x_{\min}. \end{array}$$

The exponential function is applied by (17) using the current device state in order to follow the behavior of filament growth/shrinkage. The square root for the second power of term stp(*i*_*t*_(*t*)) − *x*(*t*) is also used to keep its positive value. As analyzed in the published work [17, 23–28] examining the metal-oxide junction characteristics, it can be observed that there are numerous factors affecting the filament growth/shrinkage behavior. However there are difficulties to get experimental data about filament length and cross section area. Therefore the following constraints are introduced to facilitate modeling of these devices.

1. Filament growth occurs partially through the device cross section area [17, 35].
2. All filaments have the same length.
3. Gap permittivity and oxide permittivity are constant with respect to ions migration through the device.
4. The total cross section area of all filaments varies exponentially with tunneling current.

Hence the filament cross section area *m*(*t*)*A* can be modeled as exponentially proportional to tunneling current *i*_*t*_(*t*), where *A* is the cross section area of the junction:

(20) dmtdt=Bsstpitexp⁡⁡−E+wsittkBTexp⁡⁡−E+wgittkBTmm+Bgstp−itexp⁡⁡−E+wgittkBTfm,it,t,

(21) fm,it,t=δm−ϑmstpitt−mt2∗exp⁡⁡−Pstp−itt−mt2,

(22) $$\begin{array}{c} \delta_{m}=m_{\max}, \end{array}$$

(23) $$\begin{array}{c} \vartheta_{m}=m_{\max}-m_{\min}. \end{array}$$

As shown in Figure 6(b), (21) is used to model the exponential behavior and has to be applied in two stages using different factors (*B*_*s*1_, *B*_*g*1_, *w*_*s*1_, *w*_*g*1_, *δ*_*A*1_, *ϑ*_*A*1_ for changes from *m*_min⁡_ to *m*_max⁡⁡1_ and *B*_*s*2_, *B*_*g*2_, *w*_*s*2_, *w*_*g*2_, *δ*_*A*2_, *ϑ*_*A*2_ for changes from *m*_max⁡⁡1_ to *m*_max⁡⁡2_): the first step changes the cross section area from *m*_min⁡_ to *m*_max⁡1_ simultaneously with changing the gap thickness from *x*_max⁡_ to *x*_min⁡_, and the second step also changes the cross section area from *m*_max⁡1_ to *m*_max⁡2_ by increasing the tunneling current after the full filament growth (*x*(*t*) = *x*_min⁡_). Here *δ*_*m*1_, *δ*_*m*2_, *ϑ*_*m*1_, and *ϑ*_*m*2_ stand for *m*_max⁡1_, *m*_max⁡2_, *m*_max⁡1_ − *m*_min⁡_, and *m*_max⁡2_ − *m*_max⁡1_, respectively.

### 4.2. Capacitive Current

**Figure 7:**
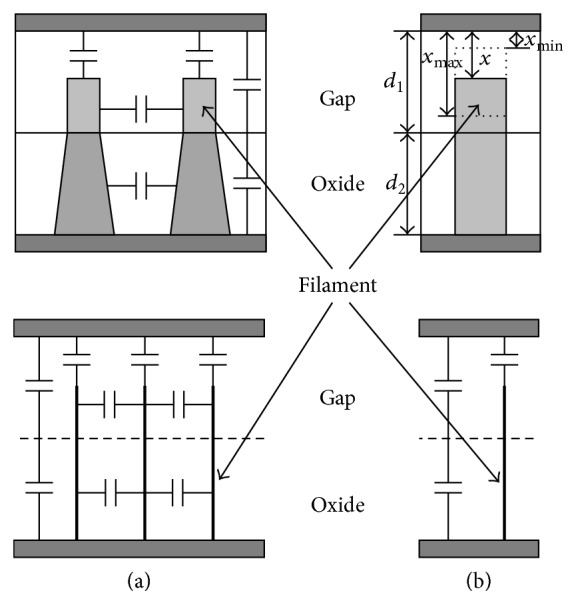
Device structure with cylindrical filaments constructed within the sandwiched layer showing (a) all capacitance components. (b) Capacitance components affecting total device capacitance.

The device capacitances can be determined by using the structure given in Figure 7 as follows.

1. Capacitances between filaments.
2. Capacitances between the top of the filaments and the upper metal.
3. Gap capacitance.
4. Oxide capacitance.

As shown in Figure 7(b), these capacitances can be reduced to three components, where the first component is short-circuited by the bottom metal. Therefore the total capacitance *C*_tot_ can be calculated as follows:

(24) $$\begin{array}{c} C_{\text{tot}}\left(t\right)=C_{\text{filament}}\left(t\right)+\frac{C_{\mathrm{gap}}\left(t\right)C_{\text{oxide}}\left(t\right)}{C_{\mathrm{gap}}\left(t\right)+C_{\text{oxide}}\left(t\right)},\\ C_{\text{filament}}\left(t\right)=\frac{A}{d_{1}}\varepsilon_{\mathrm{gap}}\frac{m\left(t\right)}{x\left(t\right)},\\ C_{\mathrm{gap}}\left(t\right)=\frac{A}{d_{1}}\varepsilon_{\mathrm{gap}}\left(1-m\left(t\right)\right),\\ C_{\text{oxide}}\left(t\right)=\frac{A}{d_{2}}\varepsilon_{\text{oxide}}\left(1-m\left(t\right)\right), \end{array}$$

where *m*(*t*)*A* represents the effective cross section area of all filaments combined together, (1 − *m*(*t*))*A* is the portion of junction cross section area that does not show any filament growth, *ε*_gap⁡_ is the gap permittivity, *ε*_oxide_ is the oxide permittivity, and *d*_1_, *d*_2_ are the gap thickness and the oxide thickness, respectively, as shown in Figure 7.

### 4.3. Tunneling Current

The tunneling current titled in the metal-oxide junctions is a function of the applied voltage [37, 38]. Figure 7(b) shows a simplified device structure which can be used to recognize the tunneling current components. Through the structure, two components of the tunneling current can be considered. The first component is between the filament and the opposite metal electrode. The second component is across the all sandwiched layers between the two metal electrodes which can be ignored because the thickness of the sandwiched materials is much larger than that of the separation between the filament and the top material. Therefore the first component is dominant component. It can be calculated using Simmons' tunneling equation between dissimilar metals as introduced by [37]. The tunneling current is a function of the applied voltage, gap thickness, and barrier heights at the interface of electrode (or filament) and gap.  Table 1 presents Simmons' tunneling equation briefly, while the appendix gives more details. This equation represents the first tunneling current component *i*_*t*_(*t*) that passes through the cross section area *m*(*t*)*A* of the filaments of the junction.

### 4.4. Model Structure

The growth/shrinkage of thin filaments depends on the tunneling current which is controlled by the applied electric field. Hence we can model the memcapacitor device with a controlled current source as shown in the previous section:

(25) $$\begin{array}{c} i\left(t\right)=i_{c}\left(t\right)+i_{t}\left(t\right), \end{array}$$

where *i*_*c*_(*t*) is the capacitive current and *i*_*t*_(*t*) is the tunneling current. Capacitive current is majored by the charge accumulation on device plates across the gap and can be calculated as follows:

(26) $$\begin{array}{c} i_{c}\left(t\right)=\dot{C_{\text{tot}}}\left(t\right)v\left(t\right)+C_{\text{tot}}\left(t\right)\dot{v}\left(t\right). \end{array}$$

The differentiation of capacitance $\dot{C_{\text{tot}}}\left(t\right)$ can be determined by measuring the current passing through a capacitance connected with a voltage source whose value equals the total capacitance of the junction *C*_tot_(*t*). The differentiation of voltage as well can be determined in the same way.

**Figure 8:**
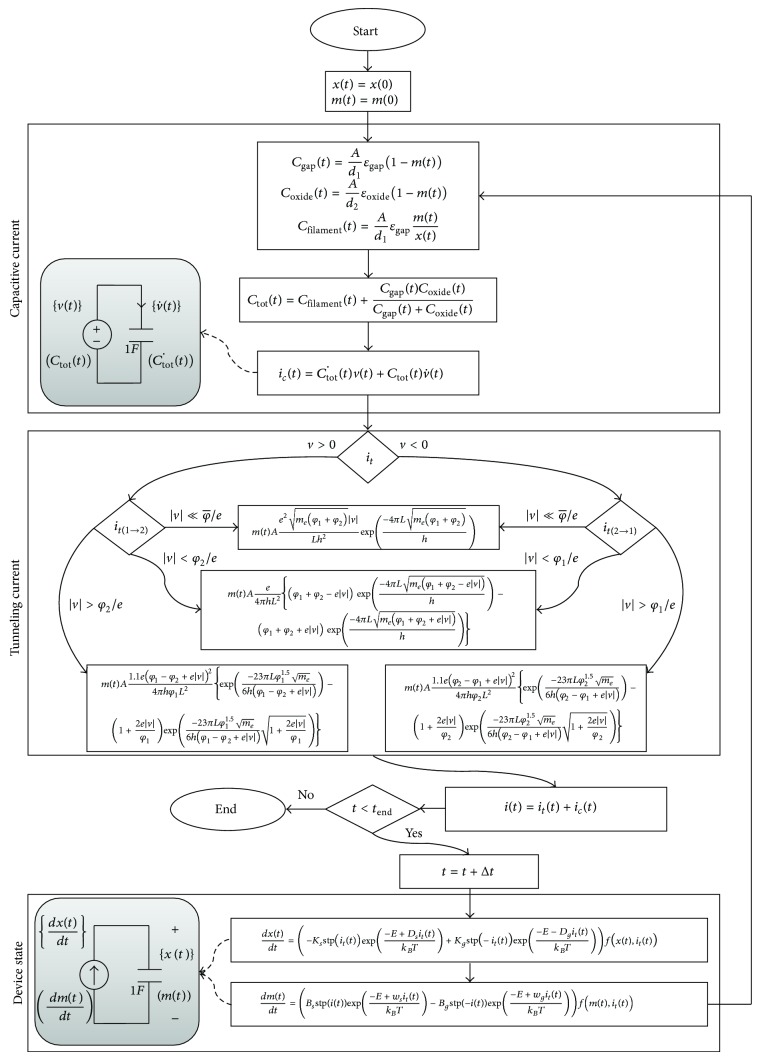
Physical modeling flow diagram of the metal-oxide junction. This diagram shows transient simulation flow through three steps of calculations. (1) Device capacitance and capacitive current. (2) Tunneling current using Simmons' tunneling equation. (3) Device state.

There are three steps for modeling of the junction as presented in the flow diagram of the junction modeling shown in Figure 8. The first step goes through device capacitance and capacitive current calculation. The second step uses Simmons' tunneling equation for tunneling current calculation. Finally the device state is calculated to be used for the next time step. We also need two integration circuits to get the values of *x*(*t*) and *m*(*t*) and two differentiation circuits to get the values of $\dot{v}\left(t\right)$ and $\dot{C_{\text{tot}}}\left(t\right)$.

In summary, thin film devices have two current components that determine the device behavior. The capacitive current component is out of phase with respect to the applied voltage because it is a function of the differentiation of the voltage as shown in (26). On the other hand the tunneling current component is in phase with respect to the applied voltage because it is a direct function of voltage as shown by Simmons' tunneling equation in Table 1. Therefor it is a resistive current. Hence the junction behavior is a combination of resistive behavior shown by tunneling current and capacitive behavior shown by capacitive current. In the next section, we will discuss what conditions make the junction show memcapacitive behavior.

## 5. Simulation Results

**Figure 9:**
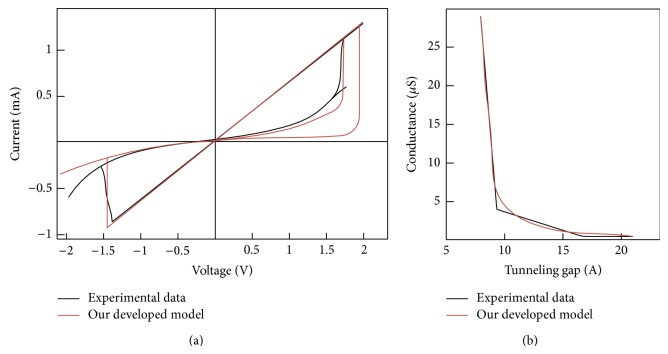
Verification of tunneling model with preciously published results. (a) A pinched hysteresis loop for a fabricated Ag (200 nm)/TiO_2−*x*_ (20 nm)/TiO_2_ (2 nm)/ITO (200 nm) memristor that shows bipolar switching behavior [39]. (b) Measured Pt/TiO_2_/Ti junction conductance versus estimated filament length using pressure-modulated conductance microscopy PCM [25]. Simulation parameter: *A* = 20*E* − 20, *P* = 5, *K*_*G*_ = 1*E*7, *K*_*S*_ = 6*E*9, *ϑ*_*x*_ = 0.4, *ϑ*_*m*_ = 0.1, *δ*_*x*_ = 0.9, *δ*_*m*_ = 0.5, *E* = 1*e* − 21, *B*_*G*_ = 9*E*6, *B*_*S*_ = 1*E*7, *D*_*G*_ = 1*E* − 14, *D*_*S*_ = 1*E* − 14, *W*_*G*_ = 1*E* − 14, *W*_*S*_ = 1*E* − 14, *φ*_1_ = *φ*_2_ = 1*e* − 19, and TEMP = 300.

As presented in the previous section, thin film device (metal-oxide junction) has two current components: tunneling current and capacitive current which express memristance and memcapacitance behaviors, respectively. These two different behaviors can be observed together or separately by choosing appropriate materials and device dimensions. Simulation results are presented to show how device parameters determine its memristance or memcapacitance behavior.  Figure 9 shows a verification of proposed model with previously published results [25, 39] which have a significant memristive behavior with unobservable capacitive behavior.

**Figure 10:**
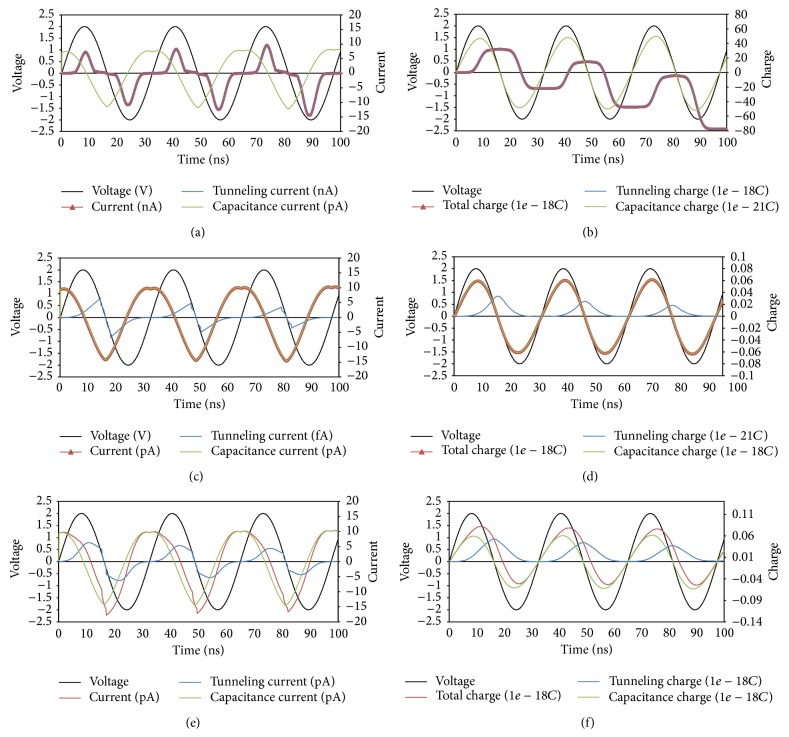
Transient simulation results of a metal-oxide junction connected with sinusoidal voltage source with peak of 2 V using the proposed physical models: (a), (c), and (e) are the results of voltage compared with current, while (b), (d), and (f) are the results of voltage compared with charge. Here (a) and (b) are the case when the tunneling current is dominant, (c) and (d) are when the capacitive current is dominant, and (e) and (f) are when tunneling current and capacitive current are comparable. Simulation parameter: *A* = 20*E* − 20, *P* = 4, *K*_*G*_ = 1*E*7, *K*_*S*_ = 6*E*9, *ϑ*_*x*_ = 0.8, *ϑ*_*m*_ = 0.8, *δ*_*x*_ = 0.9, *δ*_*m*_ = 0.9, *E* = 1*e* − 21, *B*_*G*_ = 9*E*6, *B*_*S*_ = 1*E*7, *D*_*G*_ = 1*E* − 14, *D*_*S*_ = 1*E* − 14, *W*_*G*_ = 1*E* − 14, *W*_*S*_ = 1*E* − 14, *ε*_oxide_ = 2, and TEMP = 300. The three cases are different gap characteristics where it has different permittivity in the three cases with different barrier heights. For (a) and (b) *ε*_gap⁡_ = 5, *φ*_1_ = 2.5*e* − 19, and *φ*_1_ = 1*e* − 19. For (c) and (d) *ε*_gap⁡_ = 20, *φ*_1_ = 25*e* − 19, and *φ*_1_ = 23*e* − 19. For (e) and (f) *ε*_gap⁡_ = 10, *φ*_1_ = 12*e* − 19, and *φ*_1_ = 11*e* − 19.

**Figure 11:**
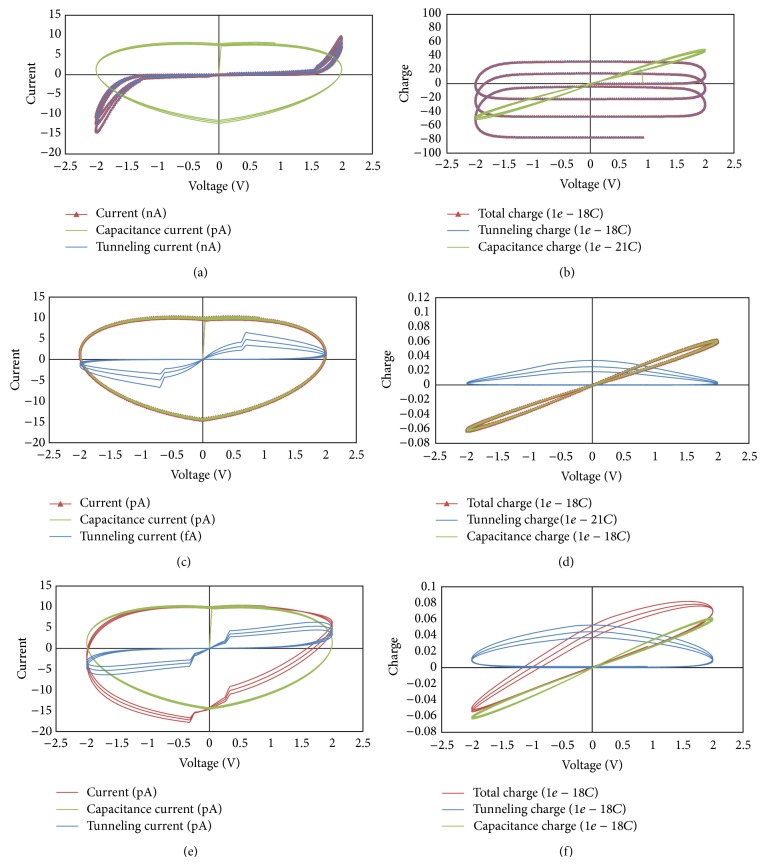
Simulation results of metal-oxide junction showing pinched hysteresis between current components and voltage: (a) when the tunneling current is dominant; (b) when the capacitive current is dominant; (c) when tunneling current and capacitive current are comparable. Simulation results of pinched hysteresis between charge components and voltage: (d) when the tunneling current is dominant; (e) when the capacitive current is dominant; (f) when tunneling current and capacitive current are comparable. Simulation parameter: *A* = 20*E* − 20, *P* = 4, *K*_*G*_ = 1*E*7, *K*_*S*_ = 6*E*9, *ϑ*_*x*_ = 0.8, *ϑ*_*m*_ = 0.8, *δ*_*x*_ = 0.9, *δ*_*m*_ = 0.9, *E* = 1*e* − 21, *B*_*G*_ = 9*E*6, *B*_*S*_ = 1*E*7, *D*_*G*_ = 1*E* − 14, *D*_*S*_ = 1*E* − 14, *W*_*G*_ = 1*E* − 14, *W*_*S*_ = 1*E* − 14, *ε*_oxide_ = 2, and TEMP = 300. The three cases are different gap characteristics where it has different permittivity in the three cases with different barrier heights. For (a) and (b) *ε*_gap⁡_ = 5, *φ*_1_ = 2.5*e* − 19, and *φ*_1_ = 1*e* − 19. For (c) and (d) *ε*_gap⁡_ = 20, *φ*_1_ = 25*e* − 19, and *φ*_1_ = 23*e* − 19. For (e) and (f) *ε*_gap⁡_ = 10, *φ*_1_ = 12*e* − 19, and *φ*_1_ = 11*e* − 19.

Figures 10 and 11 show simulations results for tunneling current, capacitive current, and total current for metal-oxide junction. Three simulation cases are considered; dominant tunneling current, dominant capacitive current and comparable current components. The tunneling current is dominant by choosing sandwiched materials with low permittivity and low barrier heights at metal/insulator interface and vice versa.  Figure 10 shows transient simulation results for current components and their first integration with time (charge) for a metal-oxide device (memristor/memcapacitor). The tunneling current component is a resistive component, while the capacitive current component is a capacitive component. This can be observed by the phase shift between the current components and the applied voltage (0° tunneling current and 90° capacitive current).

Figure 11 presents simulation results where the current and charge components show hysteresis loops with applied voltage. Three cases are presented indicating that the device works as memristor, memcapacitor, and a combination of both behaviors. Figures 11(a), 11(c), and 11(e) illustrate plots of current versus voltage that show pinched hysteresis loop when the tunneling current is dominant. From these results, we can confirm that these devices perform as memristors. Figures 11(b), 11(d), and 11(f) illustrate plots of charge versus voltage that shows other types of pinched hysteresis loops when the capacitive current component is dominant. From these results, we can conclude that these devices perform as memcapacitors.

Changing device materials has great effect on device behavior. We propose a new parameter to determine the device behavior as either memristive or memcapacitive. This parameter equals the ratio of tunneling current and capacitive current. The tunneling and capacitive current components are periodic signals with slightly variable amplitude. Hence root mean square RMS value is the best representation for current. Behavioral shape factor (BSF) is proposed parameter as follows:

(27) $$\begin{array}{c} \text{BSF}=\frac{\text{RMS}\left(i_{t}\right)}{\text{RMS}\left(i_{c}\right)}. \end{array}$$

**Figure 12:**
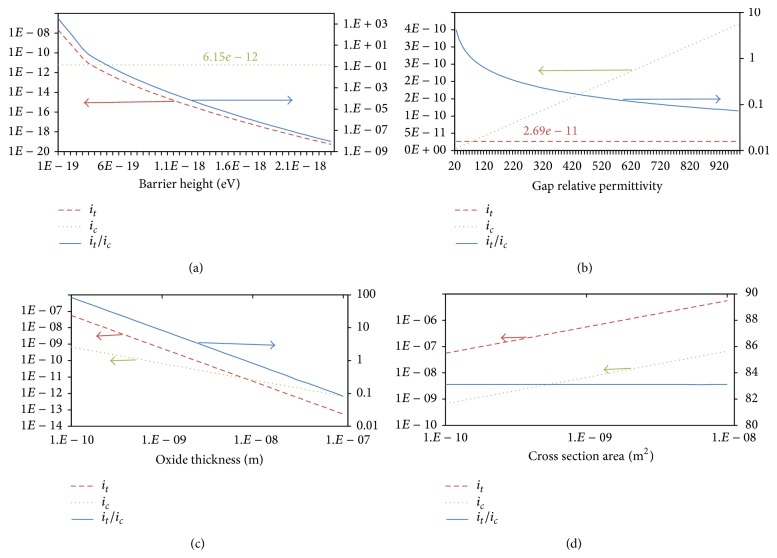
Simulation results showing the effect of changing device practical parameters (barrier height at and permittivity of sandwiched material) on device behavior plotting both current components (capacitive and tunneling) as in (a) and (c) and behavioral shape factor as in (b) and (d). Simulation parameter: *A* = 20*E* − 20, *P* = 4, *K*_*G*_ = 1*E*7, *K*_*S*_ = 6*E*9, *ϑ*_*x*_ = 0.8, *ϑ*_*m*_ = 0.8, *δ*_*x*_ = 0.9, *δ*_*m*_ = 0.9, *E* = 1*e* − 21, *B*_*G*_ = 9*E*6, *B*_*S*_ = 1*E*7, *D*_*G*_ = 1*E* − 14, *D*_*S*_ = 1*E* − 14, *W*_*G*_ = 1*E* − 14, *W*_*S*_ = 1*E* − 14, *ε*_oxide_ = 2, and TEMP = 300.

Behavioral shape factor value is greater than 0. If BSF is substantially large (e.g., BSF > 10), then the device tends to act as a memristor. However if BSF is substantially small (e.g., BSF < 0.1) then the devices tend to act as a memcapacitor.  Figure 12(a) shows the effect of changing barrier height between sandwiched material and electrodes on the root mean square value of current components.  Figure 12(b) shows BSF versus barrier height. The relation shows that changing barrier height affects greatly the tunneling current, while it has a little effect on capacitive current. Figures 12(c) and 12(d) illustrate the effect of sandwiched material's permittivity on current components and BFS. These results show that the permittivity affects only the capacitive current with no impact on the tunneling current.

## 6. Conclusions

In this paper, we discussed the possibility of building a memcapacitor device using metal-oxide structure. We developed techniques to model memcapacitors device based on the physical behavior of metal-oxide junctions. We proposed two techniques for behavioral modeling of charge-dependent and voltage-dependent memcapacitors. We then presented a physical modeling technique for metal-oxide junctions which demonstrate the combination of memristive and memcapacitive behavior, the key characteristic of such structure. Simmons' tunneling equation has been employed to model the tunneling current of the device which affects filament growth/shrinkage behavior and its capacitance change as a result. We have discovered that the barrier heights at interfaces of metal/insulator greatly affect the tunneling current, while the permittivity of sandwiched layers affects the capacitive current, and device dimensions affect both current components. Simulation results demonstrated that variation of device physical parameters has significant impact on the device behavior, consequently making the device a memcapacitor or a memristor. Behavior shape factor (BSF) has been proposed which determines device behavior as either memristive or memcapacitive. We conclude that capacitive current is dependent on tunneling current but not vice versa.

## Figures and Tables

**Figure 13:**
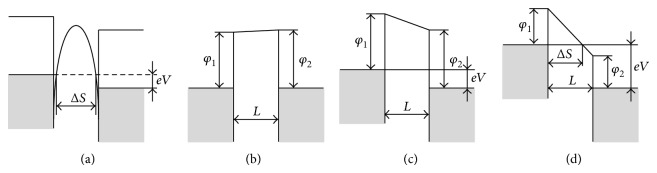
Energy band diagrams in different cases. (a) An arbitrary shape potential barrier and positive potential are applied to the right metal. (b) *V* ≈ 0. (c) *V* > *φ*/*e*. (d) *V* > *φ*/*e*.

**Table 1:** Simmons' tunneling equation.

Voltage range	Forward bias *i*_*t*(1→2)_	Reverse bias *i*_*t*(2→1)_
Low ($\left|v\right|\ll \overset{-}{\varphi }/e$)	$i_{t}\left(t\right)=\frac{e^{2}\sqrt{m_{e}\left(\varphi_{1}+\varphi_{2}\right)}\left|v\right|}{Lh^{2}}\exp\left(\frac{-4\pi L\sqrt{m_{e}\left(\varphi_{1}+\varphi_{2}\right)}}{h}\right)$	

Intermediate (|*v*| < *φ*_1_/*e* reverse bias, |*v*| < *φ*_2_/*e* forward bias)	$i_{t}\left(t\right)=m\left(t\right)A\frac{e}{4\pi hL^{2}}\left\{\left(\varphi_{1}+\varphi_{2}-e\left|v\right|\right)\exp\left(\frac{-4\pi L\sqrt{m_{e}\left(\varphi_{1}+\varphi_{2}-e\left|v\right|\right)}}{h}\right)-\left(\varphi_{1}+\varphi_{2}+e\left|v\right|\right)\exp\left(\frac{-4\pi L\sqrt{m_{e}\left(\varphi_{1}+\varphi_{2}+e\left|v\right|\right)}}{h}\right)\right\}$	

High (|*v*| > *φ*_1_/*e* reverse bias, |*v*| > *φ*_2_/*e* forward bias)	it(1→2)(t)=m(t)A1.1e(φ1-φ2+e|v|)24πhφ1L2 ×{exp⁡(-23πLφ11.5me6h(φ1-φ2+e|v|))(-23πLφ11.5me6h(φ1-φ2+e|v|)1+2e|v|φ1) -(1+2e|v|φ1)exp⁡(-23πLφ11.5me6h(φ1-φ2+e|v|)1+2e|v|φ1)}	it(2→1)(t)=m(t)A1.1e(φ2-φ1+e|v|)24πhφ2L2 ×{exp⁡(-23πLφ21.5me6h(φ2-φ1+e|v|))(-23πLφ11.5me6h(φ1-φ2+e|v|)1+2e|v|φ1) -(1+2e|v|φ2)exp⁡(-23πLφ21.5me6h(φ2-φ1+e|v|)1+2e|v|φ2)}

*m*(*t*)*A*: filament cross section area.

*e*: electron charge.

*m*_*e*_: electron mass.

*L* ^*^: barrier thickness.

*h*: Plank's constant.

*φ*_1_: barrier height at the interface of the gap and filament.

*φ*_2_: barrier height at the interface of the gap and second electrode.

^*^ *L* is equal to *x*(*t*)*d*_1_ which represents the tunneling barrier.

## Appendix

### A.

Simmons formula [37] gives an approximate expression for the tunneling current density *J* in the metal/oxide/metal junction can be written as

(A.1) J=J0φ−exp⁡⁡−Hφ−−Hφ−+eVmii−φ−+eVexp⁡⁡−Hφ−+eV,

where *J*_0_ = *e*/2*πh*(*β*Δ*s*)^2^, $H=4\pi \beta \Delta s\sqrt{2m_{e}}/h$, $\overset{-}{\varphi }$ is the mean value of the barrier height above the Fermi level, Δ*s* is barrier width, *h* is Plank constant, and *m*_*e*_ is electron mass. However *β* is a correction factor of energy band nonlinearity as shown in Figure 13(a):

(A.2) $$\begin{array}{c} \beta =1-\frac{1}{8{\overset{-}{\varphi }}^{2}\Delta s}\overset{s_{2}}{\underset{s_{1}}{\int }}{\left(\varphi \left(x\right)-\overset{-}{\varphi }\right)}^{2}dx. \end{array}$$

This current equation is valid for calculating current flow *J*_1→2_ from electrode 1 to electrode 2 (forward bias *V* > 0) and current flow *J*_2→1_ from electrode 2 to electrode 1 (reverse bias *V* < 0).

For simple calculation, trapezoidal barrier is assumed as shown in Figures 13(b), 13(c), and 13(d) with neglecting image potential. Moreover voltage range is divided into three regions.

#### A.1. Small Voltage Range

At low voltage $\left|V\right|\ll \overset{-}{\varphi }/e$, it can be considered that $\overset{-}{\varphi }$ does not depend on *V*. Therefore expression (A.1) can be simplified by substituting Δ*s* = *L* and $\overset{-}{\varphi }=\left(\varphi_{1}+\varphi_{2}\right)/2$ as shown in the energy diagram of metal/oxide/metal system for $\left|V\right|\ll \overset{-}{\varphi }/e\approx 0$ in Figure 13(b):

(A.3) J=e2meφ1+φ2VLh2×exp⁡⁡−4πLmeφ1+φ2h,

where *L* is junction thickness, *φ*_1_ is barrier height at the interface of electrode 1 with oxide material, and *φ*_2_ is barrier height at the interface of electrode 2 with oxide material.

#### A.2. Intermediate Voltage Range

As shown in Figure 13(c), at |*V*| < *φ*/*e* considering *φ* is the smallest value of (*φ*_1_, *φ*_2_), Δ*s* = *L* and $\overset{-}{\varphi }=\left(\varphi_{1}+\varphi_{2}-e\left|V\right|\right)/2$. In this case the tunneling current-voltage relation is given by

(A.4) J=e4πhL2×φ1+φ2−eV−4πLmeφ1+φ2+eVhmmi×exp⁡⁡−4πLmeφ1+φ2−eVhmmi−φ1+φ2+eVmmi×exp⁡⁡−4πLmeφ1+φ2+eVh.

This equation can be used for current flow in both forward *J*_1→2_ and reverse *J*_2→1_ directions.

#### A.3. High Voltage Range: Field Emission Mode

Case 1 . Current flow from electrode 1 to electrode 2 with |*V*| > *φ*_2_/*e* corresponds to energy diagram shown in Figure 13(d). It can be observed that Δ*s* = *Lφ*_1_/(*φ*_1_ − *φ*_2_ + *e*|*V*|) and $\overset{-}{\varphi }=\varphi_{1}/2$. Therefore, (A.1) can be rewritten as follows:

(A.5) J1→2=1.1eφ1−φ2+eV24πhφ1L2×exp⁡⁡−23πLφ11.5me6hφ1−φ2+eV−1+2eVφ1−23πLφ11.5me6hφ1−φ2+eV1+2eVφ1mmi×exp⁡⁡−23πLφ11.5me6hφ1−φ2+eV1+2eVφ1.

Case 2 . Current flow from electrode 2 to electrode 1 with |*V*| > *φ*_1_/*e* corresponds to the energy diagram shown in Figure 13(d). Parameters $\left(\Delta s,\overset{-}{\varphi }\right)$ can be considered to be Δ*s* = *Lφ*_2_/(*φ*_2_ − *φ*_1_ + *e*|*V*|) and $\overset{-}{\varphi }=\varphi_{2}/2$:

(A.6) J2→1=1.1eφ2−φ1+eV24πhφ2L2×exp⁡⁡−23πLφ21.5me6hφ2−φ1+eV−1+2eVφ2−23πLφ21.5me6hφ2−φ1+eV1+2eVφ2mmi×exp⁡⁡−23πLφ21.5me6hφ2−φ1+eV1+2eVφ2.
